# Communicating about suicide during a global pandemic: impact on journalists and media audiences

**DOI:** 10.1177/1329878X20956415

**Published:** 2021-02

**Authors:** Alexandra Wake, Elizabeth Paton, Rebecca Pryor

**Affiliations:** RMIT University, Australia; Everymind, Australia; University of Newcastle, Australia; Everymind, Australia; University of New England, Australia

**Keywords:** crisis communication, journalists, media guidelines, suicide, suicide prevention

## Abstract

COVID-19 has brought with it an increase in predictions of mental ill-health and suicide impacts in Australia. For journalists, it has been a period not only of personal stress about their economic livelihoods and occupational safety, but also balancing providing up to date information about the pandemic with safe, sensitive and accurate reporting on associated suicide and mental health issues. *Mindframe* offers guidelines, resources and individualised support to help manage the complexities of reporting on suicide in this global pandemic, working with media to protect people in their audience who are vulnerable to suicide while also helping journalists protect their own mental health and well being.

Almost as fast the COVID-19 pandemic hit the world, so did predictions of increases in mental ill-health and suicide impacts in Australia. The pandemic closely followed Australia’s devastating bushfires which raged through summer 2019/2020, taking lives and destroying homes and livelihoods. COVID-19 not only brought a health risk to the community, it also significantly impacted many of the journalists on the frontline of these stories who had barely had a moment’s rest from the bushfires. For those tasked with reporting on suicide and mental health issues, it has been a frustrating period of balancing personal worry about their economic livelihoods and occupational safety, safe and sensitive reporting, punctuated by an absence of hard statistical data for reporters, conflicting views from experts and a clear need for factual journalism to balance online misinformation. For audiences, COVID-19 has brought a similar period of personal worry as well as a surge in available information and advice, sensational headlines and problematic content that may impact on mental health and well being, particularly for those who are already vulnerable to mental ill-health or suicide. This commentary piece explores these issues and stressors for journalists and audiences, aiming to provide support that facilitates safer communication around suicide and aids both groups during the COVID-19 pandemic.

Working journalists generally know that they will at some stage put their own health at risk (Collins, 2001) on the frontline of reporting of natural disasters such as bushfires and usually are given some instruction and supported by newsrooms for such events. Peer support programmes such as those championed by the Dart Center for Journalism and Trauma (DCJT) (Asia Pacific) are well-established at the Australian national broadcaster, the Australian Broadcasting Corporation (ABC), and increasingly used in other newsrooms (Dart Centre Asia Pacific [DCAP], 2010). However, the pandemic has been different. There was no training in advance for journalists for such an event, and reporters have had to learn how to work while also social distancing, to care for themselves and their families, while watching the global death toll of journalists rise. The US-based Poynter Institute has been keeping a global list of journalists who have died as a result of the virus. That list had more than 132 names at the beginning of August 2020 (Hare, 2020). To the best of our knowledge, no Australian journalist has died of COVID-19 but the stress in Australian newsrooms has been palpable, with journalists reporting that they have had to cover street protests (Willis, 2020) and other potentially high-risk stories that others did not want to do. High-profile television Australian journalists have made a point of isolating while being tested for COVID. Channel Nine’s Richard Wilkins reported that he had contracted the illness in Australia from the US actress Rita Wilson (Cooper, 2020) and at least two others contracted the illness overseas including the *ABC*’*s* Kathryn Diss (2020) and *The Sydney Morning Herald*’*s* Bevan Shields (2020).

COVID-19 has also brought to head problems in the advertising-led business model for journalism in Australia in line with the rest of the western world putting many journalists out of work. Over 200 newspapers, radio stations and television bureaus at one point in the pandemic closed or reduced staff and services with many predicting that some will never re-open (Oderberg, 2020). Even the national broadcaster, the ABC, has had to make people redundant during this national emergency because of a funding shortfall (Meade, 2020).

Australians’ attitudes to news have changed since the beginning of the pandemic. In August, Essential Media (2020) reported that trust in the national broadcaster, the ABC, was at 58%, but news on commercial TV and radio (45%) and print media (39%) were trusted by less than half of their survey participants. Furthermore, in August, 50% agreed with the statement that they trusted the media to provide honest and objective information about the COVID-19 outbreak which was an increase from 41% in April.

An earlier study by Digital News Report Australia (Fisher et al., 2020) found that more than two-third of Australians (71%) reported they were avoiding news about the COVID-19 and this was largely driven by news fatigue. This figure was 9% higher than Australian’s usual rate of avoidance, which showed 62% of Australians avoided the news generally. Interestingly, women reported that they were more likely to avoid COVID-19 news than men because they find it upsetting. Men were more likely to avoid it because they were overwhelmed by the volume of news. That reaction is more in line with the World Health Organization’s (WHO, 2020b) concern which dubbed the problem an ‘infodemic’, or an excessive amount of information about the virus. The WHO (2020a) warned the COVID-19 ‘infodemic’ had real-world health impacts because it creates confusion and reduces the effectiveness of public health advice.

For journalists, it has been a tough job trying to present factual information to an increasingly disengaged public, with experts providing different views about the physical and psychological impact of COVID-19, and even research in the world-leading medical journal *The Lancet* being questioned and withdrawn (Davey, 2020). To support journalists, the DART Center for Journalism and Trauma produced a list of best-practice guides for covering pandemics and trauma. For journalists themselves, the DART Center recently added a list of international ‘hardship and emergency funds’ for journalists who were economically impacted by COVID-19 (DCJT, 2020), in addition to other general supports for journalists’ well being while covering trauma-related stories (DCAP, 2010; Mindframe, 2020).

COVID-19 will continue to receive heightened and ongoing media coverage and impact on journalists for some time. While providing up to date information is vitally important, so too is consideration of how media can prevent the exacerbation of fear, anxiety and mental distress within the Australian community. This is notably important for discussion of the potential impacts of COVID-19 on suicide deaths and rates, which can impact not only on the journalists covering these stories but also on audience members in the community who are already vulnerable to suicide and mental ill-health.

Suicide is a major public health issue that is affecting individuals, families, workplaces and communities across Australia. While it is common to want a simple explanation for why someone took their own life (e.g. suggesting a person died by suicide because they were socially isolated during the pandemic), ‘there is no single explanation of why people die by suicide’ (WHO, 2014: 7). Rather, the reasons for suicide are complex and multi-faceted. Each suicide death has multiple contributing factors, influenced by that person’s individual vulnerabilities and resilience as well as specific events in their life. These are combined with other social, cultural, economic and environmental factors that impact on that person and affect how they may react to life stressors.

Suicide prevention aims to decrease the number of people who die by suicide or attempt suicide each year, focusing on reducing risk factors for suicide and enhancing protective factors in conjunction with intervening as early as possible with those experiencing suicidal thoughts and behaviours. This requires action not only from within the healthcare and emergency response systems but also from all levels of government, workplaces, schools, media, communities, families and individuals. Evidence suggests that a systems or multi-component approach that addresses many of these levels is the most effective way of preventing suicide (Baker et al., 2018; Krysinska et al., 2016). One of the interventions regularly included in this systems based approach are media guidelines to ensure coverage of suicide is safe and responsible.

Ongoing media coverage at present is around a predicted increase in rates of suicide and mental illness in Australia due to the economic and social impact of COVID-19, including statements by the Victorian Coroner about higher numbers of suicide deaths in Victoria since restrictions were introduced (Silva, 2020). In early May 2020, Australian media coverage of suicide focused on modelling by The University of Sydney (2020), which predicted an additional 750–1500 suicide deaths each year for the next 5 years as an impact of COVID-19. This modelling, using a system-based approach to suicide prevention and interventions, calculated this figure based on known risk factors for suicide such as those listed above. Importantly, however, key national and international suicide prevention researchers have emphasised that these predicted deaths are *not* inevitable and focus should be on taking effective action (such as increasing health, education and social programmes) to prevent these occurring (Niederkrotenthaler et al., 2020b). This element of the research is an important message to communicate to the public, in order to prevent normalising suicide as a response to pandemic-exacerbated life stressors such as social isolation and unemployment.

Media coverage of this modelling, which the federal government’s newly appointed Deputy Medical Officer of Australia for Mental Health called ‘unwarranted at this time’ (Commonwealth of Australia, 2020), has been mixed in terms of its safety and sensitivity for people who are vulnerable to suicide. Some headlines have focused on sensational or alarmist elements. In headlines such as ‘Suicide rate could soar during coronavirus’ (AAP, 7 May 2020), ‘A new coronavirus curve “could dwarf the one we’ve been fighting”’ (Sky News, 7 May 2020) and ‘Australia’s suicide rate could surge due to economic fallout of coronavirus’ (sbs.com.au, 7 May 2020), the use of dramatic language like ‘surging’, ‘soaring’ or ‘dwarfing’ is used to attract audience attention but could misinform or alarm readers. Suicide is an emotive issue and changes in suicide statistics should be treated with care; verified, contextualised statistics and terms such as ‘increased rates’, ‘rates of concern’ or ‘significant increase are’ preferred.

Similarly, headlines such as ‘The silent COVID-19 death toll: Far more Australians will kill themselves because of coronavirus lockdown than those who die of the virus, experts say’ (Daily Mail Australia, 7 May 2020) and ‘Pandemic pain: Suicide epidemic to outstrip virus’ (The Australian, 7 May 2020) may also lead to misinform or alarm readers, with both headlines worded to suggest the inevitability of increased rates. This has the potential to normalise suicidal behaviour as a response to COVID-19–related stressors or lead to copycat or imitative behaviour in those who are already vulnerable to suicide (Bohanna and Wang, 2012; McTernan et al., 2018).

In comparison, other headlines have moderated this story with prevention or call-to-action messaging. The headline ‘“Action needed now!”: Suicide rates to exceed coronavirus deaths in Australia’ (2GB, 7 May 2020), still uses language that assumes rates of suicide will increase but places emphasis on the need for action. Taking this further, headlines such as ‘Coronavirus Australia: Suicide prevention modelling considered’ (news.com.au, 13 May 2020) and ‘Follow-up care and employment the keys to preventing many coronavirus-related youth suicides in Australia’ (abc.net.au, 14 May 2020) have avoided language that increased rates are inevitable and focused on prevention aspects of the modelling. While sharing information about the risk of increased rates is an important function of the media, it is also vital that the media emphasises that these deaths are preventable.

To ensure media reporting about suicide is safe and protective for our community, especially during this global pandemic, it is crucial that evidence around best-practice communications is taken on board. *Mindframe*, a national programme implemented by *Everymind*, provides a set of evidence-based guidelines to promote safe, sensitive and accurate portrayals of suicide in Australian media (Everymind, 2018). While there is limited evidence on the positive outcomes relating to safe media reporting on suicide, there is extensive research on reporting that is problematic or unsafe (Niederkrotenthaler et al., 2020a; Pirkis et al., 2018; WHO, 2017). This includes evidence that shows an association between problematic reporting about suicide deaths and increased rates of suicide and suicide attempts using the same method or location, and increased rates of suicide overall.

The risk of problematic media coverage increases when the reporting focuses on an individual who has died (especially celebrities), where the reporting is prominent and repeated, where the death is glamourised or glorified, and where the method and location are detailed. The *Mindframe* guidelines provide journalists with ways of minimising the risk, while still allowing for coverage of details that are within the public interest. This includes the following:

- Avoiding simplistic explanations that suggest that suicide may be the result of a single cause or factor;
- Providing context about suicide and its known risk factors;
- Framing suicide as a tragic, avoidable loss;
- Providing suicide prevention information, like warning signs and how people can access support;
- Exploring stories of those with lived experience about overcoming suicidal ideation;
- Providing help-seeking information, including 24/7 crisis support.

*Mindframe* also encourages those within the mental health or suicide prevention sectors who are interviewed as experts or writing opinion pieces to follow the *Mindframe* guidelines in their work with the media in order to ensure safer reporting on suicide.

COVID-19 brings with it an associated range of social, emotional, financial and physical challenges for all Australians and communities around the world. Journalists are among those facing increased challenges in their work life as they balance providing up to date information about the pandemic with safe, sensitive and accurate reporting on associated suicide and mental health issues. The *Mindframe* guidelines and resources are available to journalists, and the *Mindframe* team able to provide individualised support to help manage the complexities of reporting issues of suicide and mental illness, especially during this time of a global pandemic. It is important for journalists to protect their audience, particularly those who are vulnerable to suicide, while also working to protect their own mental health and well being during this period of stress and uncertainty.

If you have been impacted by these issues and are in need of immediate support, please contact Lifeline on 13 11 14 or Beyond Blue on 1300 224 636. Help is also available from your GP or regular health care provider.
